# Current management of chemotherapy-induced neutropenia in adults: key points and new challenges: Committee of Neoplastic Supportive-Care (CONS), China Anti-Cancer Association Committee of Clinical Chemotherapy, China Anti-Cancer Association

**DOI:** 10.20892/j.issn.2095-3941.2020.0069

**Published:** 2020-12-15

**Authors:** Yi Ba, Yuankai Shi, Wenqi Jiang, Jifeng Feng, Ying Cheng, Li Xiao, Qingyuan Zhang, Wensheng Qiu, Binghe Xu, Ruihua Xu, Bo Shen, Zhiguo Luo, Xiaodong Xie, Jianhua Chang, Mengzhao Wang, Yufu Li, Yuerong Shuang, Zuoxing Niu, Bo Liu, Jun Zhang, Li Zhang, Herui Yao, Conghua Xie, Huiqiang Huang, Wangjun Liao, Gongyan Chen, Xiaotian Zhang, Hanxiang An, Yanhong Deng, Ping Gong, Jianping Xiong, Qinghua Yao, Xin An, Cheng Chen, Yanxia Shi, Jialei Wang, Xiaohua Wang, Zhiqiang Wang, Puyuan Xing, Sheng Yang, Chenfei Zhou

**Affiliations:** 1 Department of Gastrointestinal Medical Oncology, Tianjin Medical University Cancer Institute and Hospital, National Clinical Research Center for Cancer, Key Laboratory of Cancer Prevention and Therapy, Tianjin, Tianjin’s Clinical Research Center for Cancer, Tianjin 300060, China; 2 Department of Medical Oncology, National Cancer Center, Cancer Hospital, Chinese Academy of Medical Sciences and Peking Union Medical College, Beijing 100021, China; 3 Department of Medical Oncology, Sun Yat-sen University Cancer Center, Guangzhou 510060, China; 4 Department of Medical Oncology, Jiangsu Cancer Hospital, Nanjing 210009, China; 5 Department of Oncology, Jilin Province Cancer Hospital, Changchun 130012, China; 6 Department of Oncology, Zhongshan Hospital Affiliated to Xiamen University, Xiamen 361004, China; 7 Department of Oncology, Cancer Hospital Harbin Medical University, Harbin 150081, China; 8 Department of Oncology, Affiliated Hospital of Qingdao University, Qingdao 266003, China; 9 Department of Medical Oncology, Fudan University Shanghai Cancer Center, Shanghai 200032, China; 10 Department of Oncology, General Hospital of Shenyang Military Region, Shenyang 110016, China; 11 Department of Pulmonary and Critical Care Medicine, Peking Union Medical College Hospital, Beijing 100730, China; 12 Department of Hematology, Affiliated Cancer Hospital of Zhengzhou University, Henan Cancer Hospital, Zhengzhou 450008, China; 13 Lymphoma and Myeloma Department, Jiangxi Cancer Hospital, Nanchang 330029, China; 14 Department of Medical Oncology, Shandong Cancer Hospital, Shandong Academy of Medical Sciences, Jinan 250117, China; 15 Department of Oncology, Ruijin Hospital, Shanghai Jiao Tong University School of Medicine, Shanghai 200025, China; 16 Sun Yat-sen Memorial Hospital, Sun Yat-sen University, Guangzhou 510120, China; 17 Department of Radiation and Medical Oncology, Zhongnan Hospital of Wuhan University, Wuhan 430070, China; 18 Department of Oncology, Nanfang Hospital, Southern Medical University, Guangzhou 510515, China; 19 Department of Gastrointestinal Oncology, Peking University Cancer Hospital & Institute, Beijing 100142, China; 20 Department of Medical Oncology, Xiang’an Hospital of Xiamen University, Xiamen 361101, China; 21 Department of Medical Oncology, The Sixth Affiliated Hospital, Sun Yat-sen University, Guangzhou 510655, China; 22 Department of Oncology, The First Affiliated Hospital, Shihezi University School of Medicine, Shihezi 832000, China; 23 Department of Oncology, The First Affiliated Hospital of Nanchang University, Nanchang 330006, China; 24 Department of Integrated Chinese and Western Medicine, Cancer Hospital of University of Chinese Academy of Science, Zhejiang Cancer Hospital, Hangzhou 310022, China

**Keywords:** Chemotherapy-induced neutropenia (CIN), febrile neutropenia, cancer, risk stratification, granulocyte-colony stimulating factor (G-CSF)

## Abstract

Chemotherapy-induced neutropenia (CIN) is a potentially fatal and common complication in myelosuppressive chemotherapy. The timing and grade of CIN may play prognostic and predictive roles in cancer therapy. CIN is associated with older age, poor functional and nutritional status, the presence of significant comorbidities, the type of cancer, previous chemotherapy cycles, the stage of the disease, specific chemotherapy regimens, and combined therapies. There are many key points and new challenges in the management of CIN in adults including: (1) Genetic risk factors to evaluate the patient’s risk for CIN remain unclear. However, these risk factors urgently need to be identified. (2) Febrile neutropenia (FN) remains one of the most common reasons for oncological emergency. No consensus nomogram for FN risk assessment has been established. (3) Different assessment tools [e.g., Multinational Association for Supportive Care in Cancer (MASCC), the Clinical Index of Stable Febrile Neutropenia (CISNE) score model, and other tools] have been suggested to help stratify the risk of complications in patients with FN. However, current tools have limitations. The CISNE score model is useful to support decision-making, especially for patients with stable FN. (4) There are still some challenges, including the benefits of granulocyte colony stimulating factor treatment and the optimal antibiotic regimen in emergency management of FN. In view of the current reports, our group discusses the key points, new challenges, and management of CIN.

## Introduction

Chemotherapy-induced neutropenia (CIN) is a major cause of hematological and dose-limiting toxicities of chemotherapy. It may have short- or long-term impacts on treatment plans, which may result in unfavorable disease control and survival. Patients may also miss potential opportunities to be cured due to the severe consequences of CIN. CIN and/or febrile neutropenia (FN) often result in high costs, severe infections, aggressive hospital management, life-threatening morbidity, and even mortality. This review article focuses on the key points of managing CIN, including but not limited to the pathogenesis, risk factors, predictive risk models, and available strategies regarding the optimal management of CIN.

## Definition and grading system of CIN

CIN is generally characterized as a decreased absolute neutrophil count (ANC) < 2,000 cells/mm^3^ in peripheral blood. Further classification of the severity of CIN is evaluated by the National Cancer Institute Common Terminology Criteria for Adverse Events, version 5.0. According to this grading system, neutropenia is classified according to the following 4 grades: (i) Grade 1 with an ANC of 1,500–2,000 cells/mm^3^, (ii) Grade 2 with an ANC of 1,000–1,500 cells/mm^3^, (iii) Grade 3 with an ANC of 500–1,000 cells/mm^3^, and (iv) Grade 4 with an ANC < 500 cells/mm^3^.

## Impacts of CIN on the clinical outcomes of cancer patients

Several studies have shown that CIN may predict favorable prognoses and therapeutic results in some cancers. These studies showed that CIN may be associated with better survival [progression-free survival (PFS) and/or overall survival (OS)] or a lower recurrence risk in breast cancer^1^, gastric cancer^2^, cervical cancer^3^, pancreatic cancer^4^, lung cancer^5^, colorectal cancer^6^, and ovarian cancer patients^7^.

A new viewpoint on the timing of CIN as a prognostic and predictive factor in cancer patients has been suggested in several studies^7–9^. In advanced pancreatic cancer, the timing of CIN is an independent prognostic factor for those patients treated with gemcitabine monotherapy or gemcitabine-based combination chemotherapy, and patients with early onset CIN (EOCIN) showed better median overall survival (mOS) than those patients without EOCIN (mOS: 8.05 months *vs.* 5.82 months,* P* = 0.028)^8^. In metastatic colon cancer, the timing of CIN is an independent prognostic factor for patients who were treated with mFOLFOX6, and patients with EOCIN also showed better OS and PFS than those patients without EOCIN (OS: 18.5 months *vs.* 9.5 months, *P* < 0.001; PFS: 7.1 months *vs.* 3.4 months, *P* < 0.001)^9^. In advanced gastric cancer, EOCIN may also be a potential prognostic factor for patients treated with capecitabine plus oxaliplatin as the first-line chemotherapy, and patients with EOCIN showed significantly better mOS and PFS than those patients without EOCIN (OS: 16.7 months *vs.* 12.8 months, *P* < 0.001; PFS: 8.3 months *vs.*6.3 months, *P* < 0.001)^10^. Additionally, in metastatic non-small cell lung cancer patients, the timing of CIN is also a prognostic factor for patients treated with gemcitabine combined with cisplatin chemotherapy. A previous study showed that patients with EOCIN showed better mOS and PFS than those patients with late onset CIN (LOCIN) or those without CIN (mOS: 16.7 months *vs.* 11.2 months, *P* = 0.0004; PFS: 5.1 months *vs.* 3.8 months, *P* = 0.0016)^11^.

Whether high grade CIN can be used to predict favorable survival outcomes remains controversial in clinical practice. Some studies have reported that compared with patients with mild/moderate CIN (grades 1–2), patients with severe CIN (grades 3–4) have improved OS. However, the prognostic roles of CIN grades are different in different cancers and chemotherapies. The reason may be that body surface area-based dose calculations cannot fully consider the individual differences in drug metabolism. The prognostic role of the CIN grade will be further elaborated based on the study of tailored drug metabolism.

## Biomarkers of the risk and incidence of developing CIN

All patients undergoing chemotherapy have a risk of developing CIN. CIN is specifically associated with older age, poorer functional and nutritional status, the presence of significant comorbidities, certain types of cancer, previous chemotherapy cycles, disseminated diseases, particular chemotherapy regimens, and combined therapy. Cancer patients with diabetes mellitus or hyperglycemia have a 32% higher chance of developing CIN than patients without these two conditions^12^.

Predicting and screening high risk CIN patients is of great clinical significance. Such advances may help to monitor and manage CIN and improve the outcomes of chemotherapy. However, biomarkers to predict and screen for the risk of CIN are still unavailable. The SUCCESSA trial showed that hyaluronan-mediated motility receptor (HMMR) gene single nucleotide polymorphisms (SNPs) are significant predictors of CIN in female patients with breast cancer who underwent FEC chemotherapy^13^. A meta-analysis revealed that SLCO1B1 521T>C or 1118G>A can predict a 2- to 4-fold increased risk for irinotecan-induced CIN in East Asian patients^14^. UGT1A1^*^93 and SLCO1B1^*^1b were found to be new predictors of irinotecan-related neutropenia^15^. In the TOSCA randomized trial from Italy with regimens of FOLFOX4 or XELOX as adjuvant chemotherapy for colorectal cancer, DPYD variants (^*^6 rs1801160 and ^*^2A rs3918290) were significantly associated with time to neutropenia^16^. In castration-resistant prostate cancer, age and baseline ANC were independent predictors for severe CIN (grade 4) induced by a docetaxel-based regimen^17^. Furthermore, rs1453542 in OR4D6 can be used as a new biomarker to predict the risk for CIN induced by GC (gemcitabine plus carboplatin) in non-small cell lung cancer^18^.

## FN and risk assessment

The definition of FN is ≥ 38.3 °C orally or ≥ 38.0 °C for a duration of over 1 h. Occurrences of FN will prompt major life-threatening complications and lead to oncological emergency. A series of risks, including high morbidity, mortality, cost and dose reductions, and chemotherapy delays may accompany FN. In solid tumors, the incidence of FN is 13%–21% for common myelosuppressive chemotherapy. Usually, the incidence of FN occurring during the first cycle of chemotherapy is much higher (23%–36%)^19^. However, in the real world, the incidence of FN is much higher than the percentage reported from randomized controlled trials.

Evaluation of the FN risk should be performed before initial chemotherapy. Patients can then be classified into 3 risk levels according to the intensity of the chemotherapy regimen and the characteristics of the patients. The classification of FN is as follows: high risk FN with risk > 20%, intermediate risk FN with risk 10%–20%, or low risk FN with risk < 10%.

Before each subsequent cycle, the FN risk should be evaluated to help clinicians make treatment decisions, including FN risk categorization and chemotherapy intent. To date, no consensus has been reached on FN risk assessment based on clinical evidence. However, guidelines or consensuses from different regions in the world recommend that the overall risk of FN should take into account both the chemotherapy regimens and patient characteristics.

### Patient-related risk factors for FN

Before we assess the risk factors for FN, we should distinguish the risk factors for FN and the patient-related FN risk factors that promote serious complications and mortality. This concept is important not only for those patients with high risk FN, but also for those patients with low/intermediate risk FN. **Table 1** shows patient-related risk factors for FN^20^. **Table 2** shows the risk factors that promote serious complications and mortality.

**Table 1:** Patient-related risk factors for FN

Risk Factors for FN					
•	Older age (≥ 65 years)	•	Anemia (Haemoglobin <12 g/dL)	•	Baseline ANC < 1500 cells/mm^3^
•	Advanced disease/metastasis	•	Cardiovascular disease	•	Baseline serum albumin ≤ 3.5 g/dL
•	No antibiotic prophylaxis	•	Renal disease	•	Poor nutritional status and/or lower weight
•	Prior episode of FN	•	Abnormal liver transaminases	•	Prior chemotherapy and radiotherapy
•	No use of G-CSFs	•	ECOG score ≥ 2	•	Prior infection
•	Female	•	Patient with comorbidity (≥ 1)		
•	Asian race				

**Table 2:** The risk factors for the patients with febrile neutropenia (FN) that promote serious complications and mortality

Risk Factors for the patients with FN to promote serious complications and mortality			
•	Sepsis syndrome	•	Invasive fungal infection (IFI)
•	Age > 65 years	•	Infections caused by other pathogens
•	Severe neutropenia (ANC < 100 cells/mm^3^)	•	Time to antibiotics (TTA)
•	Neutropenia expected to be > 10 days in duration	•	Previous FN
		•	Pneumonia

### Chemotherapy regimen-related risk factors for FN

In addition to considering patient-related factors, the chemotherapy regimen should also be considered as an important risk factor for FN. The overall risk factors should be combined to determine whether to treat with preventive intervention using G-CSF. In chemotherapy-naïve patients, the incidence of FN induced by chemotherapy regimens is > 20%, which is considered a high risk chemotherapy regimen, while an incidence of 10%–20% is considered an intermediate risk, and an incidence lower than 10% is considered a low risk in clinical trials. **Table 3** shows the FN risk categories for common chemotherapy regimens^19–22^.

**Table 3:** The risk categories of chemotherapy regimen to induce febrile neutropenia (FN)

Cancer type	FN risk category (%)/Chemotherapy regimen		
	< 10	10–20	> 20
Breast cancer	AC	FEC/docetaxel	AC- docetaxel
	Epirubicin/cyclophosphamide ± lonidamide	FEC-120 FEC-100	Docetaxel-AC
	Doxorubicin/cyclophosphamide–paclitaxel	Cyclophosphamide/mitoxantrone	Doxorubicin/docetaxel
	CMF	Paclitaxel (every 21 days)	Doxorubicin/paclitaxel
	Doxorubicin/cyclophosphamide	DDG doxorubicin/Cyclophosphamide-paclitaxel	TAC TCH
	FAC 50	Doxorubicin/vinorelbine	
		AC	
Small cell lung cancer	CAV - PE	Etoposide/carboplatin	ACE
		CAV	Topotecan
		Etoposide/carboplatin	ICE
		Paclitaxel/carboplatin	VICE
		Tirapazamine/cisplatin/etoposide/irradiation	DDG CAV -PE
		CODE	
Non-small cell lung cancer	Gemcitabine/cisplatin	Paclitaxel/cisplatin	Docetaxel/carboplatin
		Vinorelbine/cisplatin	
		Paclitaxel/carboplatin	
		Cisplatin/docetaxel	
		Etoposide/cisplatin	
		Docetaxel	
Non-Hodgkin lymphoma		ACOD	DHAP
		(R)-CHOP	ESHAP
		Fludarabine/mitoxantrone	R-ESHAP
		Dose adjusted EPOCH	
		Mega dose-CHOP	VAPEC-B
		(R)-GEM-P	ACVBP
		(R)-GEMOX (elderly patients)	(R)-Hyper-CVAD
		GDP	ICE/R-ICE
		CHP	Stanford V
			MOPPEB-VCAD
			FC
			FCR
			
Hodgkin’s disease			BEACOPP
			ABVD
			CEC
			IGEV
Ovarian cancer	Gemcitabine/cisplatin	Paclitaxel/carboplatin	Docetaxel
			Topotecan
Urothelial cancer		Paclitaxel/carboplatin	MVAC
			DDGc MVAC
			BOP--VIP-B46
Germ cell tumours		Cisplatin/etoposide	VeIP
		BEP - EP	
Colorectal cancer	Irinotecan	FOLFOX	
	IFL	FOLFIRI	
Gastric cancer		Docetaxel-irinotecan	DCF
		FOLFOX	TC
		LVFU-cisplatin	TCF
		LVFU-irinotecan	ECF
			ECX
			EOF
			EOX
Esophagal cancer		Irinotecan/cisplatin	
Other malignancies	Doxorubicin/cisplatin (endometrial cancer)	Gemcitabine/irinotecan (pancreatic cancer) FOLFIRINOX (pancreatic cancer)	TIC (head and neck cancers)
	TAP (endometrial cancer)	Stanford V (Hodgkin’s lymphoma)	MAID (sarcoma)
	TPF (laryngeal cancer)	Paclitaxel/cisplatin (cervical cancer)	
		Gemcitabine/docetaxel (occult primary- adenocarcinoma)	

DD, dose-dense; DDG, dose-dense with G-CSF; AC, Cyclophosphamide+Adriamycin; FEC, Epirubicin+Cyclophosphamide+Fluorouracil; CMF, Methotrexate+Cyclophosphamide+Fluorouracil; TAC, Docetaxel+Epirubicin+Cyclophosphamide; TCH, Docetaxel+carboplatin+trastuzumab; ACE, Etoposide+Epirubicin+Cyclophosphamide; CAV, Vincristine+Etoposide+Epirubicin; PE, Etoposide+Cisplatin; ICE, Ifosfamide+Epirubicin+ Cyclophosphamide; VICE, Ifosfamide+carboplatin+etoposide+vincristine; CODE, Vincristine+Etoposide+Cisplatin+Epirubicin; CHOP, cyclophosphamide++vincristine+doxorubicin+ponisone; GDP, gemcitabine+dexamethasone+cisplatin/carboplatin; CHP, cyclophosphamide+doxorubicin,+prednisone; DHAP, cisplatin+cytarabine+dexamethasone; ESAP, cytarabine+etoposide+ 6-mercaptopurine+cisplatin; ABVD, doxorubicin+bleomycin+vinblastine+dacarbazine; BEACOPP, etoposide+doxorubicin+ cyclophosphamide+vincristine+bleomycin+prednisone+procarbazine; EPOCH, etoposide+vincristine+cyclophosphamide+ doxorubicin+prednisone; StanfordV, doxorubicin+vincristine+nitrogenmustard+vinblastine+bleomycin+etoposide+prednisone; MAID, mesner+doxorubicin+ifosfamide+dacarbazine; IGEV, Isophosphoramide+gemcitabine+vinorelbine+prednisone; FOLFOX, oxaliplatin+fluorouracil+calciumleucovorin; FOLFIRI, Irinotecan+fluorouracil+calciumleucovorin; DCF, Docetaxel+cisplatin+fluorouracil; TCF, Taxol+cisplatin+fluorouracil; ECF, Epirubicin+cisplatin+fluorouracil; EOF, Epirubicin+oxaliplatin+ fluorouracil; EOX, Epirubicin+oxaliplatin+capecitabine; ECX, Cisplatin+capecitabine+epirubicin; BEP, Bleomycin+etoposide+cisplatin; TPF, Taxol+cisplatin+fluorouracil; FOLFIRINOX, Irinotecan+oxaliplatin+fluorouracil + calcium leucovorin.

## Assessment tools for risk stratification and a prognostic model for FN

FN is a diverse syndrome. Some assessment tools, including the Talcott model, MASCC risk index, and CISNE model, have been tested and verified to assess the risk of FN in clinical trials. However, compared with the Talcott and MASCC systems, the FINITE study showed that the CISNE model is effective and accurate for classifying stable FN episodes despite their heterogeneities^23^. Receiver operating characteristic analysis showed that the area under the characteristic (AUC) curve for the Talcott model was 0.652, that for the MASCC model was 0.721, and that for the CISNE model was 0.868 (*P* = 0.002, for the CISNE model *vs.* the MASCC model).

### MASCC risk index

**Figure 1:**
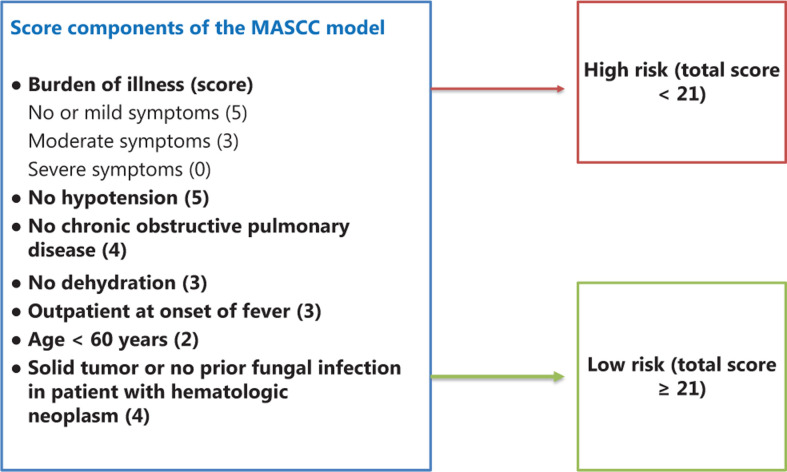
The Multinational Association for Supportive Care in Cancer (MASCC) scoring system. The components of the MASCC have a maximum score of 26 (5 + 5 + 4 + 4 + 3 + 3 + 2). Patients with scores ≥ 21 are at low risk for complications, while patients with scores < 21 are at high risk for complications.

The MASCC model was published in 2000 and has been widely used in recent decades to stratify the risk of FN. The MASCC scoring system divides patients with FN into 2 risk groups (low and high risk for complications) and can predict FN complications. It can be used as a basic tool for clinicians to select overall care and treatment strategies. **Figure 1** shows the components of the MASCC^24^, with a maximum score of 26 (5 + 5 + 4 + 4 + 3 + 3 + 2). Patients with scores ≥ 21 are at low risk for complications, while patients with scores < 21 are at high risk for complications. However, some studies showed the limitations of the MASCC score, especially in specificity and outpatient management. The MASCC model cannot specifically identify patients who may actually have a risk of complications and who should be hospitalized for FN^25^.

### Talcott classification system

For outpatient therapy, the Talcott model divides patients with FN into 4 groups according to the risk of complications: 1st group, the FN complication rate is 34%, and outpatients are hospitalized with fever and neutropenia; 2nd group, the FN complication rate is 55%, and outpatients are hospitalized with comorbidities; 3rd group, the FN complication rate is 31%, and outpatients are hospitalized without comorbidities but with uncontrolled cancer conditions; and 4th group, the FN complication rate is 2%, and outpatients are hospitalized without comorbidities and with uncontrolled cancer conditions^26,27^. The Talcott model has limitations, with a high misclassification rate (59%) and low sensitivity (30%)^26^.

### CISNE model

**Figure 2:**
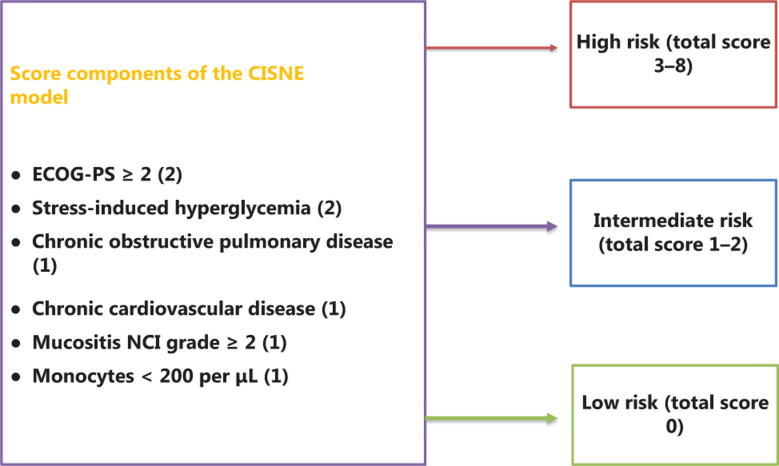
The Clinical Index of Stable Febrile Neutropenia (CISNE) scoring system. The components of the CISNE have a maximum score of 8 (2 + 2+ 1 + 1 + 1+ 1). Patients with a score of 0 are at low risk for complications, patients with a score of 1–2 are at intermediate risk for complications, and patients with a score of 3–8 are at high risk for complications.

The CISNE model (**Figure 2**)^23,28^ is a predictive tool for FN patients who seem to be initially stable but subsequently develop serious complications. The CISNE scoring system has convenient online calculators that are accessible on computers or mobile phones to provide risk assessment (https://www.mdcalc.com/clinical-index-stable-febrile-neutropenia-cisne). The CISNE calculator (available for Android and as an iOS app) is a tool that is easy to use and estimates and suggests the actions and most necessary approaches for management. The CISNE model is especially useful for outpatient management to identify those patients with low risk FN complications. It can also be considered the most appropriate FN risk-stratification tool in the emergency room. The MASCC model is much less effective in identifying low risk patients than the CISNE model. The overall risk score for the CISNE model is divided into three prognostic categories: low risk (0 points), intermediate risk (1–2 points), and high risk (≥ 3 points). With a cutoff of ≥ 3 points, the CISNE model shows good discriminatory power to predict major complications^23^. This critical cutoff (≥ 3 points) is an important layered basis for subsequent treatment decisions. However, the CISNE model is not applicable for patients with unstable FN, serious infections, lymphoma, or hematological malignancies.

### PROMASCC model

A prognostic model was developed based on patient-reported outcomes (PROs). The PROMASCC model was established to identify FN patients with a low risk of developing complications. By incorporating the Functional Assessment of Cancer Therapy-Neutropenia (FACT-N) elements, the PROMASCC model aggregates the psychometric properties of the FACT-N and MASCC scores to improve the evaluation performance. The FACT-N subscales consist of 5 core parts: physical subscales, functional subscales, social/family subscales, emotional subscales, and a 19 item subscale. A single center, cross-sectional observational study revealed that psychological evaluation of patient self-discomfort can improve the stratified assessment of FN^29^. In the PROMASCC scoring system, univariate and multivariate analyses determines individual weights, and the scoring system ranges from 0–3. The scoring system shows a MASCC risk index ≥ 21 (score = 2) and a Malaise Subscale score ≥ 11 (score = 1). The PROMASCC model has more advantages than the MASCC model, including better specificity (64.3% *vs.* 38.1%), a higher positive predictive rate (81.0% *vs.* 73.7%), a lower misclassification rate (24.2% *vs.* 25.8%), and an increase in the AUC (0.732 *vs.* 0.658)^29^.

### A new prognostic model of FN from the Republic of Korea

A new prognostic model of FN from the Republic of Korea that classifies and assesses patients for increased adverse outcomes and bacteremia has been reported. Risk factor weights in this new model are as follows: age ≥ 60 years counts as 2 points; procalcitonin ≥ 0.5 ng/mL counts as 5 points; performance score ≥ 2 counts as 2 points; mucositis grade ≥ 3 counts as 3 points; SBP < 90 mmHg counts as 3 points; and RR ≥ 24 breaths/minute counts as 3 points^30^. The model classifies patients with FN into 3 groups: the low risk group (score ≤ 2) with an incidence of adverse outcomes of 6.0% and of bacteremia of 1.1%; the intermediate-risk group (scores 3–8) with an incidence of adverse outcomes of 27.3% and bacteremia of 11.5%; and the high risk group (score ≥ 9) with an incidence of adverse outcomes of 67.9% and bacteremia of 29.8%^30^. Low risk patients can be discharged early, intermediate risk patients can be managed with short-term observation, and high risk patients should be managed with inpatient therapy. This tool aims for higher specificity and negative predictive values. It is a simple and objective risk stratification tool. However, a limitation of this tool is that the validation set is from a single center, nonrandom study, which might affect its representativeness.

### Other valuable risk factors of FN

Procalcitonin (PCT) is an independent predictor for serious complications of FN. Serum PCT levels can be established as a risk assessment tool similar to the MASCC model. The combination of C-reactive protein (CRP) and the MASCC model was successful in assessing the mortality risk of patients with FN and hematological malignancies. Multivariate analysis showed that MASCC scores and CRP levels were independent survival risk factors. The 30 day survival rate for those patients with a low risk MASCC score and low CRP level (≤ 15 mg/dL) was 100%. However, the percentage for a high risk MASCC score and high CRP level (> 15 mg/dL) was only 64%^31^. This assessment has a potential role in predicting the risk of death within the first 5 days of FN.

## Recommendations

- FN patients can develop serious complications and often need to be cared for as an emergency with hospitalization. Different assessment tools have been established to stratify the risk of complications in FN patients. Based on these stratifications, subsequent interventions and treatments are determined. However, every tool has its specific limitations.
- The MASCC model is challenged by its unsatisfactory specificity, and its universal application has gradually been reduced. The combination of the MASCC score with other biochemical biomarkers and/or new models is critical to improving sensitivity and specificity when deciding the personalized treatments for FN patients.
- The CISNE model is a good FN risk-stratification tool for emergency practice. It is also appropriate for outpatient treatment to identify low risk FN patients. The CISNE calculator (available for Android and as an iOS app) is an easy and useful tool that estimates and suggests actions and most necessary approaches for emergency treatments.

## Risk factors for patients with FN that predict poor outcomes

The treatment of FN includes two important aspects: (1) stratifying the risk of complications in FN patients, and (2) predicting poor outcomes in FN patients. Platelet count (< 50,000 cells/mm^3^), protein level (< 6 g/dL), respiratory rate (> 24/min), pulmonary infiltration, serum CRP (> 50 mg/dL), MASCC score (< 21), and eGFR (≤ 90 ML/min/1.73 m^2^) have been reported to be independent predictors of poor outcomes for patients with FN^32^. Moreover, it is worth noting that 4 factors (platelet count, pulmonary infiltration, hypoproteinemia, and respiratory rate) are especially important for predicting FN mortality.

## Management of CIN

In the management of CIN, myeloid growth factors (MGFs), including G-CSF and granulocyte macrophage-colony stimulating factor (GM-CSF), are approved for clinical practice to reduce the risk of FN and its complications. Primary G-CSF prophylaxis can significantly reduce the risk of FN based on evidence from 17 RCTs that enrolled 3,500 patients with solid tumors or lymphomas^33^. However, there is insufficient evidence to suggest that MGFs can increase the survival of patients (disease-free survival and/or overall survival). Moreover, MGFs have economic cost and some potential risks, such as a risk for inducing acute myeloid leukemia or myelodysplastic syndromes. Therefore, FN risk assessment is very important, and it is an important basis for weighing the advantages and disadvantages of using MGFs.

According to the action time, MGFs are classified into two types: long-acting (pegfilgrastim, lipegfilgrastim, etc.), and short-acting (filgrastim, lenograstim, etc.). Compared with short-acting drugs, long-acting drugs have similar effects, with the advantage of being more convenient to use (a single injection 24–72 h after chemotherapy) and a disadvantage of high cost.

## Prophylactic treatment with G-CSF for the prevention of FN

The clinician should assess the patient’s overall risk of developing FN before every chemotherapy cycle. Four important factors (patient-related risk factors, chemotherapy regimen, complications, and treatment intent) must be comprehensively considered to evaluate the overall FN risk. Patients classified with high FN risk (≥ 20%) should be recommended for prophylactic use of G-CSF. Patients classified with intermediate FN risk (10%–20%) should be considered for prophylactic use of G-CSF, and a comprehensive discussion of the risk-benefit ratio should occur between the patient and doctor. For patients classified with low FN risk (< 10%), prophylactic use of G-CSF usually is not recommended^34^.

## Recommendations

**Figure 3:**
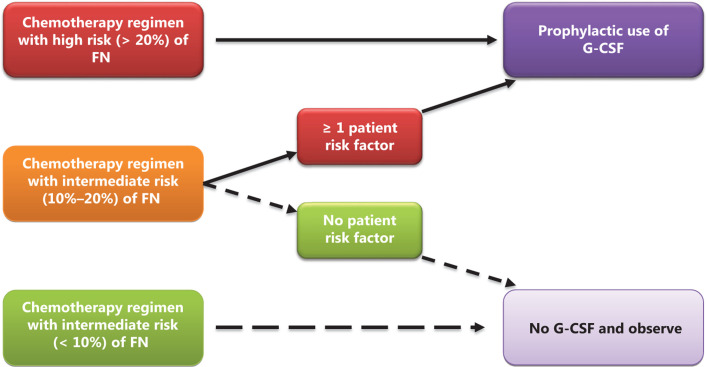
Recommendations for the prophylactic use of G-CSF based on chemotherapy regimens and patient risk factors. FN, febrile neutropenia, G-CSF, colony-stimulating factor.

- Each patient’s overall FN risk should be evaluated before every cycle of chemotherapy. Primary and secondary CSF prophylaxis should be recommended differently according to the patient’s overall risk. The assessment should include type of disease, treatment intent, chemotherapy regimen, dose (**Table 3**) and patient risk factors (**Table 1**).
- Prophylactic use of G-CSF should be considered if the chemotherapy regimen has a high risk of inducing FN.
- If the chemotherapy regimen is classified as having an intermediate risk of FN, the prophylactic use of G-CSF should be considered based on the patient’s risk as follows: 1) patients with at least 1 risk factor (**Table 1**) should consider receiving prophylactic G-CSF; and 2) patients with no risk factors do not need prophylactic use of G-CSF and observation (**Figure 3**).
- If the chemotherapy regimen is classified as having a low risk of FN, the patient does not need prophylactic G-CSF and observation (**Figure 1**).
- Patients who had suffered from prior FN or CIN who do not experience a chemotherapy delay or dose reduction are classified as having a high risk of FN for the current cycle. If a patient still develops FN after prophylactic use of G-CSF, clinicians should consider reducing the dose or changing the regimen in the next chemotherapy cycle unless an adjustment would affect patient survival.

## Guidance recommendations for prophylactic short-acting G-CSF and long-acting G-CSF for FN

Short-acting G-CSF is used commonly to prevent CIN/FN and is the main part of G-CSF’s clinical application. Long-acting G-CSF is increasingly used due to its advantage of easy administration (one subcutaneous administration for every cycle). Prophylactic G-CSFs are effective and safe for preventing CIN/FN if they are initiated 24~72 h after chemotherapy^35^. However, for patients receiving a 14 day chemotherapy regimen, the initial dose of prophylactic G-CSFs should be received specifically at day 7 after the initiation of chemotherapy. Prophylactic G-CSF should only be administered 3–4 times for a 14-day chemotherapy regimen.

PEG-G-CSFs are preferred by patients and physicians for favorable safety and efficacy. In clinical trials and practice, prophylactic PEG-G-CSFs can reduce the risks of FN and FN-related complications more than short-acting G-CSFs^36,37^. Importantly, PEG-G-CSFs are more convenient than short-acting G-CSFs due to the one-per-cycle administration.

## Recommendations

- Prophylactic PEG-G-CSFs facilitates a single administration and may be preferred to the administration of short-acting G-CSFs to prevent FN and FN-related complications, especially for old or frail patients.
- Without sufficient clinical trial evidence, prophylactic PEG-G-CSFs are not recommended for patients undergoing weekly or metronomic chemotherapy.
- In patients undergoing split-dose chemotherapy, PEG-G-CSF administration is recommended 24 h after the last dose of chemotherapy.
- Once initiated, it is suggested that prophylactic PEG-G-CSFs be continuously administered throughout all cycles of chemotherapy^38^. Data from multiple reports support the administration of PEG-G-CSFs at least 1 day after chemotherapy^35,38–41^.

## Guidance recommendations for the therapeutic use of G-CSFs for FN

It is worth noting that there is some evidence, though not sufficient evidence, to support the therapeutic use of G-CSFs for FN. The use of G-CSFs could reduce the incidence of severe CIN, the rate of hospitalization, and the use of antibiotics. However, there is no evidence to support these benefits improving the OS of patients.

## Recommendations

- FN patients with prophylactic short-acting G-CSFs should be continued by administering the same G-CSFs^21,42^.
- FN patients with prophylactic long-acting G-CSFs (PEG-G-CSFs) should not be treated with additional short-acting G-CSFs^38,43^.

For FN patients without prophylactic G-CSFs, clinicians must carefully assess the risk of FN patient complications and poor prognoses. In particular, infection-related complications should be the focus of assessments. Risk factor assessment should include age > 65 years, sepsis syndrome, ANC < 100 cells/mm^3^, duration of CIN > 10 days, pneumonia, evidence of infection (especially invasive fungal infections), hospitalization, and previous FN^44–46^. If FN patients have any of these risk factors, G-CSFs are recommended.

## Treatment for FN

**Figure 4:**
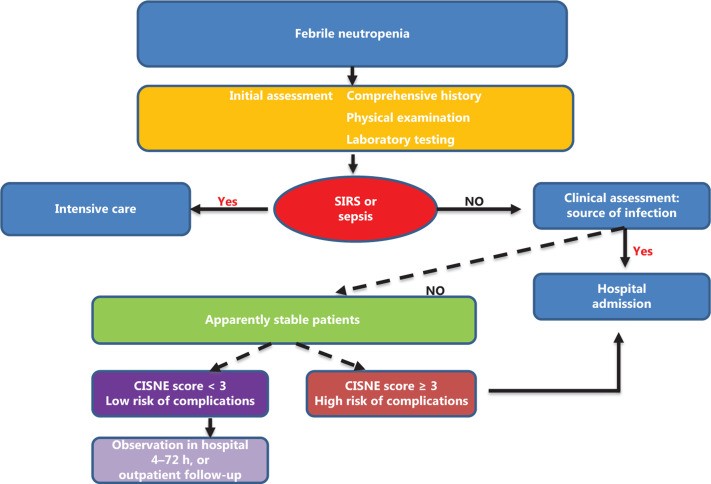
Decision-making algorithm in febrile neutropenia according to the CISNE score. CISNE, clinical index of stable febrile neutropenia; SIRS, systemic inflammatory response syndrome.

Before FN treatment, especially before empirical broad-spectrum antimicrobial therapy, detailed and comprehensive assessment needs to be performed. First, an initial evaluation should be performed, including a comprehensive history and physical examination, with further testing using blood counts, microbiological cultures, and radiographical measurements. Second, it is recommended that clinicians determine treatment decisions for patients with stable FN based on the CISNE model (**Figure 4**). Patients with a high risk of FN complications (CISNE score ≥ 3) should be hospitalized. The discharge criteria for high risk patients should meet two important points: the blood culture is negative, and the patient is identified as having stable FN. However, for those patients with low/intermediate risk of FN complications (i.e., a CISNE score 0–2), coinfections and clinical risk factors must be excluded^28^.

The therapeutic benefit of G-CSFs for FN treatment remains controversial. The role of G-CSFs plus antibiotics does not affect the overall mortality of FN patients, especially infection-related mortality. However, G-CSFs could accelerate the recovery of FN, shorten the hospital stay of patients, and reduce the use of antibiotics.

The potential sites, pathogens causing infection, the patient’s risk of developing infection-related complications, and the laboratory/radiology results should be evaluated for FN patients before empirical antimicrobial therapy. The potential sites of infection commonly include the alimentary tract, skin, lungs, sinus, and ears, and the perivaginal/perirectal, urological, neurological, and intravascular access device sites^47^. The spectrum of pathogenic bacteria in different infection sites is significantly different. The pathogens causing infection for FN patients include Gram-negative bacteria, Gram-positive bacteria, viruses, and fungi. Common Gram-negative bacteria include Escherichia coli, Klebsiella pneumoniae, copper Pseudomonas aeruginosa, Stenotrophomonas maltophilia, and Acinetobacter baumannii. Common Gram-positive bacteria include Staphylococcus epidermidis, enterococci (including vancomycin-resistant enterococcus), streptococcus, Staphylococcus aureus [including methicillin-resistant Staphylococcus aureus (MRSA)], and coagulation solid enzyme-negative staphylococci^48^. In principle, antibiotics should be used after the microbial culture results are obtained. However, without early use of antibiotics, patients with FN could progress rapidly and develop fatal complications. The principle for empirical antimicrobial therapy is to consider the most common and virulent pathogenic bacteria that can cause serious complications or death until accurate pathogenic culture results are obtained. The optimal antibiotic regimen for these bacteria is still controversial. Moreover, the empirical use of antibiotics could induce antibiotic resistance (including MRSA or drug-resistant Gram-negative bacteria)^49^.

## Recommendations

- Patients with a high risk of FN complications (CISNE score ≥ 3) should be hospitalized and treated with intravenous broad-spectrum antibiotics. Initial empirical antibiotic application should be considered to treat Pseudomonas aeruginosa and other serious Gram-negative pathogens. First-line drugs are recommended, such as antipseudomonal β-lactam-cefepime (2 g IV every 8 h), piperacillin tazobactam (3.375 g IV every 6 h for mild-moderate infections, or 4.5 g IV every 6–8 h for severe infections), carbapenem-imipenem (500 mg IV every 6 h) or meropenem (1–2 g IV every 8 h or 500 mg IV every 6 h)^50^.
- In patients with a low risk of FN complications (CISNE score 0–2), oral empirical antibiotic treatment should be considered for selected patients. The following first-line drugs for oral empirical antibiotic application are recommended: fluoroquinolones (moxifloxacin 400 mg PO/IV daily or levofloxacin 500–750 mg oral or IV daily), monotherapies or ciprofloxacin (500–750 mg PO every 12 h or 400 mg IV every 8–12 h) plus amoxicillin-clavulanate (875 mg PO every 12 h)^51–53^.
- For Gram-positive bacteria, susceptibility to vancomycin has decreased. Linezolid (600 mg PO/IV every 12 h) and daptomycin (6 mg/kg/d IV) are active agents and options for initial empirical treatment^54^. Treatment with these agents should be based on anti-infection guidelines, the antibacterial spectrum, and adverse reactions.
- For empirical or preemptive antifungal treatment, the evidence is limited. Empirical antifungal treatment can be initiated if FN patients remain febrile or have recrudescent fever after 72–96 h of broad-spectrum antibiotic treatment. Caspofungin (70 mg IV × 1 dose, then 50 mg IV daily) and liposomal amphotericin B (3–5 mg/kg IV daily) might be used for empirical treatment^47^. However, stable FN patients without evidence of invasive fungal disease (determined by a lung computed tomography scan, measurement of circulating Aspergillus galactomannan antigen, and clinical examination) might not need empirical anti-fungal treatment.
